# Clinicopathological characteristics, prognosis, and the significance of FNA-CT in sporadic medullary thyroid microcarcinoma: a 10-year retrospective study

**DOI:** 10.3389/fendo.2025.1676241

**Published:** 2025-10-15

**Authors:** Mingjun Wang, Wenjie Chen, Peiheng Li, Yanping Gong

**Affiliations:** Division of Thyroid Surgery, Department of General Surgery, West China Hospital, Sichuan University, Chengdu, Sichuan, China

**Keywords:** sporadic medullary thyroid microcarcinoma (micro-sMTC), calcitonin assays in fine-needle aspiration washout fluid (FNA-CT), dynamic risk stratification, lymph node metastasis (LNM), progression-free survival (PFS)

## Abstract

**Objective:**

The detection rate of sporadic medullary thyroid microcarcinoma (micro-sMTC) has increased with advancements in diagnostic techniques. In this study, we aimed to investigate the clinical characteristics, optimal management, prognosis, and the significance of calcitonin assays in fine-needle aspiration washout fluid (FNA-CT) for micro-sMTC, which remain unclear.

**Methods:**

This retrospective study included 73 patients with micro-sMTC who underwent initial surgery between November 2014 and November 2024 at the Division of Thyroid Surgery, Department of General Surgery, West China Hospital, Sichuan University. Clinicopathological features, factors associated with lymph node metastasis (LNM), dynamic risk stratification, and progression-free survival (PFS) were analyzed. Additionally, the significance of FNA-CT was investigated in relation to early diagnosis, surgical decision-making, and prognosis in these patients.

**Results:**

The mean age of the patients was 48.3 years, with a female predominance (60.3%). Following the introduction of FNA-CT in 2020, the detection rate of micro-sMTC increased. Most patients (91.8%) presented with asymptomatic suspicious thyroid nodules detected on ultrasonograms. The median tumor size and basal calcitonin level were 7.0 mm and 68.0 pg/mL, respectively. FNAC accurately identified MTC in only 20 (24.4%) of 82 nodules from the 73 patients. Hemithyroidectomy was performed in 13 (17.8%) patients, including 4 with contralateral nodules. After a median follow-up of 34.0 months (range: 6.0–124.0 months), 58 (79.5%) patients achieved an excellent response, while 12 (16.4%) and 3 (4.1%) patients exhibited biochemical incomplete and structural incomplete responses, respectively. LNM was significantly associated with suspicious lymph nodes on ultrasonograms, multifocality, and high-grade tumors. High-grade histology and advanced clinical stage were associated with an unfavorable response to initial surgery and independently predicted poor PFS (both *p* < 0.05). Patients in the FNA-CT group had lower basal calcitonin and carcinoembryonic antigen (CEA) levels, smaller tumors, less advanced staging, and fewer high-grade tumors (all *p* < 0.05) than those in the non-FNA-CT group. However, early diagnosis via FNA-CT was not associated with improved PFS.

**Conclusions:**

Despite its typically favorable prognosis, micro-sMTC may exhibit certain aggressive features. High tumor grade and advanced clinical stage are independent prognostic factors of poor PFS and may guide postoperative surveillance strategies. FNA-CT shows potential value in facilitating earlier diagnosis of micro-sMTC and identifying suitable candidates for hemithyroidectomy.

## Introduction

Medullary thyroid carcinoma (MTC) is a rare, calcitonin-secreting neuroendocrine tumor, accounting for less than 5% of all thyroid cancers, however, it represents up to 13% of thyroid-cancer-related deaths (1, 2). Approximately 75% of MTCs are sporadic (sMTC), while the remaining 25% are hereditary (hMTC). Based on the size, MTC is traditionally classified into medullary thyroid microcarcinoma (micro-MTC, maximum diameter ≤1 cm) and medullary thyroid macrocarcinoma (macro-MTC, maximum diameter >1 cm) (3). Owing to its occult nature, most micro-MTCs are incidentally diagnosed following thyroidectomy for other thyroid pathologies (4, 5).

The widespread use of high-resolution neck ultrasonography, calcitonin testing, calcitonin assays in fine-needle aspiration washout fluid (FNA-CT), and the increasing thyroidectomy rates have contributed to an increasing trend in the detection of micro-MTC (6–9). However, the biological characteristics, natural history, and clinical management of micro-MTC remain largely unknown, as findings from previous studies have been conflicting. Kim et al. reported that patients with macro-MTC were more likely to have lymph node metastasis (LNM) than those with micro-MTC, however, both Aubert and Kesby failed to confirm this association (5, 10, 11). Saltiki et al. observed that patients with micro-MTC of ≤5 mm had a less advanced stage at diagnosis than those with micro-MTC of 6–10 mm (12). Furthermore, Machens et al. reported that incidentally detected unifocal micro-MTC of ≤5 mm following hemithyroidectomy did not require further completion thyroidectomy or lymph node dissection (LND), which was consistent with the findings of Gui Z’s study (13, 14). However, other studies have demonstrated that micro-MTC can exhibit aggressive characteristics similar to macro-MTC, including LNM, multifocality, extrathyroidal extension (ETE), and distant metastasis (10, 15).

Although several guidelines regarding the diagnosis and management of MTC have been established by various countries and professional associations, guidelines for managing micro-MTC remain controversial. The Japanese Association of Endocrine Surgeons (JAES) recommends hemithyroidectomy for selected patients with sporadic medullary thyroid microcarcinoma (micro-sMTC), and the National Comprehensive Cancer Network states that central LND is not mandatory for such cases (16, 17). In contrast, other guidelines recommend total thyroidectomy (TT) with central LND for the management of MTC (1, 18–21). As surgery is currently the only curative option for MTC, the early diagnosis and appropriate management of micro-MTC may improve patient prognosis. Our recent and others’ previous studies have demonstrated that FNA-CT is a beneficial and cost-effective method for the early detection of occult MTC (22, 23). However, its impact on clinical decision-making and long-term prognosis in patients with micro-sMTC remains unclear.

Owing to the rarity of micro-sMTC, ongoing controversy surrounding its management, and unclear clinical utility of the FNA-CT, we conducted this retrospective study to assess the clinicopathological features and long-term outcomes of surgically treated patients with micro-sMTC at our center. Additionally, we aimed to investigate the significance of FNA-CT in the early diagnosis, therapeutic decision-making, and prognosis of micro-sMTC.

## Materials and methods

A total of 89 consecutive patients with micro-MTC who underwent initial surgery between November 2014 and November 2024 at the Division of Thyroid Surgery, Department of General Surgery, West China Hospital, Sichuan University, were initially enrolled in the study. After surgery, patients were suggested for germline genetic testing. Exclusion criteria were as follows: 1) hMTC diagnosis based on family history and/or germline pathogenic variants in the REarranged during Transfection (RET) proto-oncogene; 2) patients with incomplete clinical and pathological data; and 3) follow-up time of less than 6 months. This study was approved by the ethics committee of West China Hospital, Sichuan University. Informed consent was obtained from all patients before surgery.

Data were obtained from a prospectively maintained and regularly updated clinical database at West China Hospital, Sichuan University. The data collected for analysis included patient demographics (age at diagnosis, sex, year of diagnosis, and comorbidities), initial clinical presentation, preoperative laboratory tests (basal calcitonin, basal carcinoembryonic antigen [CEA] levels), preoperative fine-needle aspiration cytology (FNAC) and FNA-CT, surgical details (initial surgical approach and LND extent), final pathological results (pathological diagnosis, lymph node status, tumor extension, tumor foci, tumor grade, and tumor stage), postoperative complications, and follow-up information (dynamic risk stratification, follow-up time, recurrence, and mortality).

Owing to the 10-year duration of the study, management strategies of micro-sMTC in our center have evolved. Before 2020, preoperative diagnosis of micro-sMTC was primarily based on preoperative ultrasonography, basal calcitonin, basal CEA, and FNAC results, excluding patients with family history and/or germline pathogenic variants in the RET proto-oncogene. Since 2020, FNA-CT has been introduced as an auxiliary tool to enhance the preoperative detection of MTC (23). Therefore, the study period was treated as a categorical variable and divided into two intervals: 2014–2019 and 2020–2024. The largest tumor diameter was measured using preoperative ultrasonography and recorded as both a continuous and a binary variable (1–5 mm and 6–10 mm). In principle, patients with a preoperative confirmed micro-sMTC were treated with TT and bilateral central LND. Lateral LND was performed based on preoperative ultrasonography, FNAC and/or FNA-CT results of suspicious lateral lymph nodes, or basal calcitonin levels greater than 200 pg/mL. Owing to the increased early detection rate of micro-sMTC with the use of FNA-CT and the favorable prognosis of this cancer, hemithyroidectomy with ipsilateral central LND was considered as an alternative option for patients with solitary sMTC lesion of ≤5 mm in maximum diameter, clinically negative lymph nodes (cN0), and lesion not adjacent to the thyroid capsule on preoperative ultrasonogram. Before surgery, these patients were thoroughly informed about the recurrence risk of hemithyroidectomy, the potential for conversion to TT based on intraoperative findings, and the possibility of requiring completion thyroidectomy during follow-up. The final decision on whether to undergo TT or hemithyroidectomy was made at the discretion of the surgeon and the patient. The International Medullary Thyroid Carcinoma Grading System (IMTCGS) was introduced in 2022; in the preceding years, the pathology slides were independently reviewed by two pathologists who were blinded to patients’ clinical and previous pathological results to evaluate the tumor grade. In case of discrepancies, the slides were simultaneously reviewed by the two pathologists to establish a final consensus. From 2022 onward, tumor grade was routinely provided in a structured pathological report. Tumors were defined as high-grade based on the presence of tumor necrosis, a mitotic index ≥ 5 per 2 mm², or Ki-67 proliferation index ≥ 5% (24). The tumor stage was determined according to the American Joint Committee on Cancer (AJCC) Cancer Staging Manual, 8th edition (25). FNAC results were based on the morphological characteristics of MTC and reported according to the Bethesda System for Reporting Thyroid Cytopathology (TBSRTC I-VI) (26, 27).

Postoperative complications, including hypoparathyroidism, recurrent laryngeal nerve injury, chylous leakage, and Horner syndrome, were recorded. Hypoparathyroidism was categorized as transient or persistent forms, which were defined as postoperative hypocalcemia with a low or inappropriately normal parathyroid hormone level that resolved within 6 months, and hypocalcemia and/or the continued need for calcium and/or active vitamin D supplementation over 6 months following surgery, respectively. Recurrent laryngeal nerve injury was also classified as transient or persistent, based on recovery within 6 months after surgery.

Patients returned to our outpatient clinic for follow-up at 1 and 3 months postoperatively. Subsequently, follow-up was conducted every 6 months during the first 3 years, and annually thereafter, either at our hospital or at a local hospital. Calcitonin measurement and neck ultrasonography were routinely performed to evaluate responses to initial surgery. Additional follow-up and imaging studies, such as CT or PET-CT, were performed when clinically indicated. The follow-up data can be obtained from the clinical database or Huayitong app, an official medical application released by the West China Hospital of Sichuan University, which provides online consultation, health advice, and follow-up services to users (28). Response to initial surgery, referred to as dynamic risk stratification, was categorized as excellent response, biochemical incomplete response, and structural incomplete response. Following the American Thyroid Association (ATA) recommendations, serum calcitonin levels measured 3 months postoperatively were used to assess responses. To ensure the accuracy of the analysis, patients with persistent biochemical or structural disease, defined as calcitonin levels not returning to the normal range and/or structural disease identified on imaging or biopsy within 3 months after surgery, were excluded. Additionally, patients using medications known to interfere with calcitonin levels, such as omeprazole, beta-blockers, and glucocorticoid secretagogues, were also excluded. Therefore, excellent response was defined as undetectable calcitonin and CEA within the reference ranges and no structural evidence of disease throughout follow-up. Biochemical incomplete response was defined as newly detectable calcitonin or elevated CEA levels without structural disease, while structural incomplete response referred to newly identified structural disease via imaging or biopsy more than 3 months after surgery.

**Figure 1:**
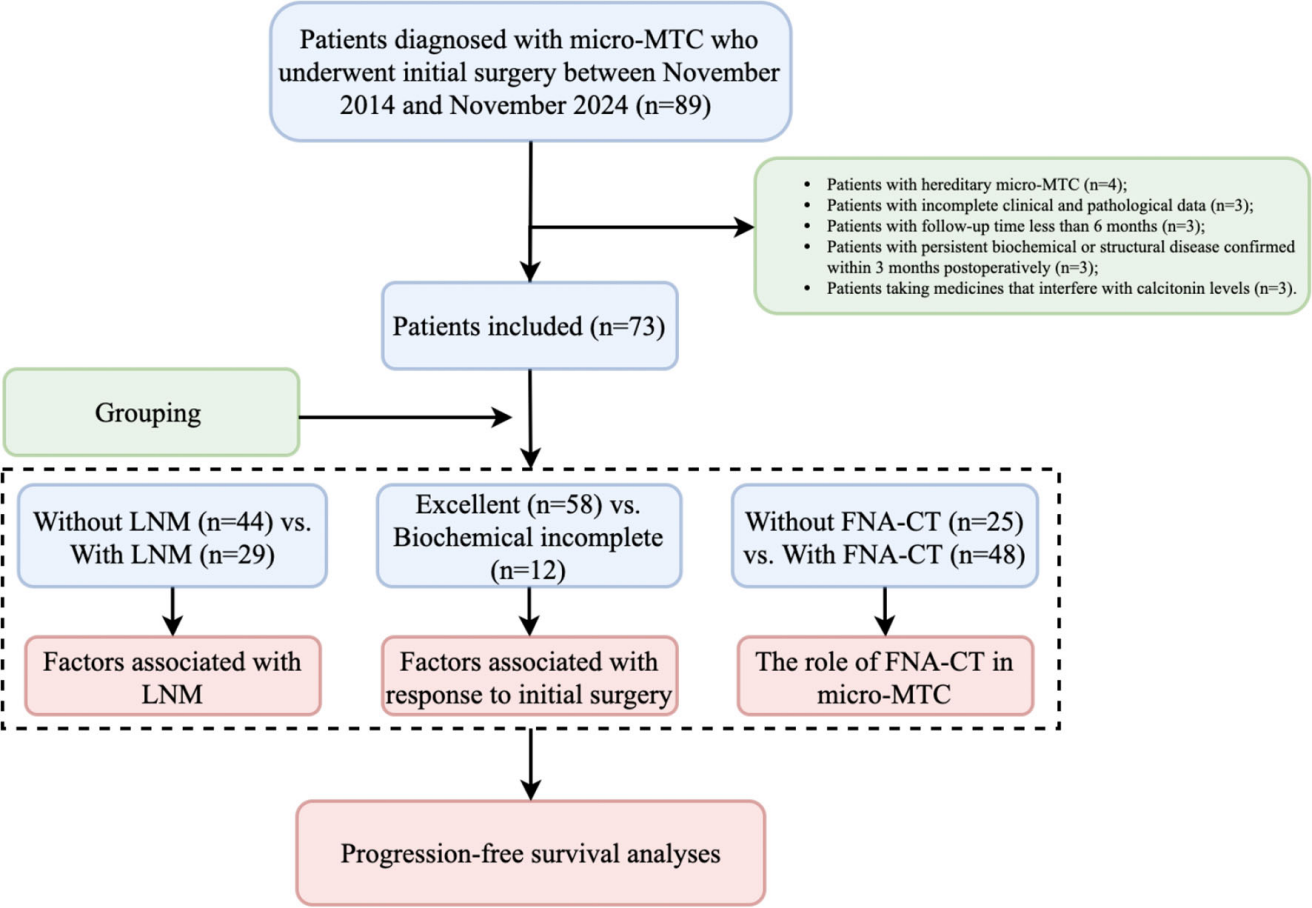
Study flowchart. micro-MTC, Medullary thyroid microcarcinoma; LNM, Lymph node metastasis; FNA-CT, Calcitonin assays in fine-needle aspiration washout fluid.

Patients were allocated into groups based on LNM, response to initial surgery, and the utility of FNA-CT. Factors associated with LNM and dynamic risk stratification were analyzed, and the impact of FNA-CT in the early diagnosis, management, and prognosis of micro-sMTC was assessed. Owing to the very low incidence of structural incomplete response in patients with micro-sMTC, biochemical incomplete response was designated as the primary endpoint. The most recent follow-up was completed in May 2025, ensuring a minimum follow-up duration of 6 months. To avoid immortal-time bias, progression-free survival (PFS) was defined as the period from 3 months postoperatively to the diagnosis of biochemical incomplete response. A brief flowchart of the study is illustrated in **Figure 1**.

### Statistical analyses

Quantitative data were expressed as mean ± standard deviation for normally distributed variables and as median values with ranges for skewed quantitative data. Categorical variables were summarized as frequencies with corresponding percentages. All statistical analyses, including Student’s *t*-test, Mann–Whitney *U* test, chi-square test, Fisher’s exact test, univariate and multivariate Cox proportional hazards regression, and Kaplan–Meier survival analysis with the log-rank test, were performed using R software (version 4.4.2). A two-sided *p*-value < 0.05 was considered statistically significant.

## Results

### Characteristics of the study participants

A total of 73 patients with micro-sMTC were included in the study (**Table 1**). The mean age of these patients was 48.3 ± 11.7 years, with 44 (60.3%) being women. Sixty-two patients (84.9%) were diagnosed after 2020, whereas only 11 patients (15.1%) were identified before 2020. Most patients (91.8%) were asymptomatic and were found to have suspicious thyroid nodules with or without nodular goiters, during routine ultrasonography screening. The median basal calcitonin and CEA values were 68.0 pg/mL (range: 12.5–7080.0 pg/mL) and 3.3 ng/mL (range: 0.7–291.0 ng/mL), respectively. FNA-CT was performed in 48 patients (65.8%), and the median FNA-CT value from 53 micro-MTC lesions among these patients was 34222.0 pg/mL (range: 3453.0–200000.0 pg/mL). FNAC was performed on 82 nodules from 73 patients: 20 (24.4%) nodules were accurately diagnosed as MTC, whereas 10 (12.2%), 4 (4.9%), 43 (52.4%), and 5 (6.1%) nodules were classified as TBSRTC I, II, III, and IV, respectively. The median tumor size was 7.0 mm (range: 3.0–10.0 mm), with 50 patients (68.5%) having a tumor size greater than 5.0 mm. Thirteen patients (17.8%) chose hemithyroidectomy as their initial surgical approach and lateral LND was performed in 17 patients (23.3%). Final pathological results revealed that 13 patients (17.8%) exhibited ETE, 8 patients (11.0%) had multifocal micro-MTC, and 29 patients (39.7%) had LNM. High-grade micro-MTC was identified in 17 patients (23.3%). Additionally, coexisting thyroid carcinomas were observed in a few cases, including 3 patients (4.1%) with papillary thyroid carcinoma, 2 patients (2.7%) with the follicular variant of papillary thyroid carcinoma, and 1 patient (1.4%) with noninvasive follicular thyroid neoplasm with papillary-like nuclear features. According to the pathological findings, 44 patients (60.3%) were classified as AJCC clinical stage I, 21 patients (28.8%) as stage III, and 8 patients (11.0%) as stage IV. Postoperative complications were detected in 34 cases, all of whom were managed conservatively. After a median follow-up of 34.0 months (range: 6.0–124.0 months), 58 patients (79.5%) achieved an excellent response, whereas 12 (16.4%) and 3 (4.1%) patients exhibited biochemical and structural incomplete responses, respectively. All patients survived throughout the follow-up period.

**Table 1:** Characteristics of the study participants.

Variables	Values
Age at diagnosis (years)	48.3 ± 11.7
Sex	
Female	44 (60.3%)
Male	29 (39.7%)
Year of diagnosis	
2014–2019	11 (15.1%)
2020–2024	62 (84.9%)
Comorbidities	
HT	11 (15.1%)
Diabetes	4 (5.5%)
CVD	8 (11.0%)
Others	5 (6.8%)
Initial presentation	
Asymptomatic suspicious thyroid nodule detected using US	67 (91.8%)
Enlarged cervical lymph nodes	5 (6.8%)
Hyperthyroidism	1 (1.4%)
Basal calcitonin level (pg/mL)	68.0 (12.5–7080.0)
Basal CEA level (ng/mL)	3.3 (0.7–291.0)
FNA-CT cases	48 (65.8%)
FNA-CT value ^a^ (pg/mL)	34222.0 (3453.0–200000.0)
Suspicious lymph nodes identified using preoperative US	6 (8.2%)
FNAC ^b^	
I	10 (12.2%)
II	4 (4.9%)
III	43 (52.4%)
IV	5 (6.1%)
V–VI	20 (24.4%)
Tumor size (mm)	7.0 (3.0–10.0)
Tumor size category (mm)	
1.0–5.0	23 (31.5%)
6.0–10.0	50 (68.5%)
Tumor extension	
Intrathyroid	60 (82.2%)
ETE	13 (17.8%)
Foci	
Solitary	65 (89.0%)
Multifocal	8 (11.0%)
Lymph node status	
N0	44 (60.3%)
N1	29 (39.7%)
Distant metastasis	
M0	73 (100.0%)
M1	0
Final pathology	
MTC	67 (91.8%)
MTC+PTC	3 (4.1%)
MTC+FVPTC	2 (2.7%)
MTC+NIFTP	1 (1.4%)
AJCC clinical stage	
I	44 (60.3%)
III	21 (28.8%)
IV	8 (11.0%)
Tumor grade	
Low	56 (76.7%)
High	17 (23.3%)
Initial surgical approach	
Hemithyroidectomy	13 (17.8%)
Total thyroidectomy	60 (82.2%)
LND extent	
Central LND	56 (76.7%)
Central and Lateral LND	17 (23.3%)
Postoperative complications	
Transient hypoparathyroidism	30 (41.1%)
Transient RLN injury	2 (2.7%)
Permanent hypoparathyroidism	0
Permanent RLN injury	0
Chylous leakage	1 (1.4%)
Horner syndrome	1 (1.4%)
Follow-up duration (months)	34.0 (6.0–124.0)
Response to initial surgery	
Excellent	58 (79.5%)
Biochemical incomplete	12 (16.4%)
Structural incomplete	3 (4.1%)
Death	0

micro-sMTC, Sporadic medullary thyroid microcarcinoma; HT, Hashimoto thyroiditis; CVD, Cardiovascular diseases; US, Ultrasonography; CEA, Carcinoembryonic antigen; FNA-CT, Calcitonin assays in fine-needle aspiration washout fluid; FNAC, Fine-needle aspiration cytology; ETE, Extrathyroidal extension; MTC, Medullary thyroid carcinoma; PTC, Papillary thyroid carcinoma; FVPTC, Follicular variant of papillary thyroid carcinoma; NIFTP, noninvasive follicular thyroid neoplasm with papillary-like nuclear features; AJCC, American Joint Committee on Cancer; LND, Lymph node dissection; RLN, Recurrent laryngeal nerve.

^a^Data from 53 micro-MTC nodules of 48 patients; ^b^Data from 82 nodules of 73 patients.

### Factors associated with LNM and dynamic risk stratification

As presented in **Table 2**, patients with LNM had higher basal calcitonin levels (140.3 pg/mL, range: 13.1–7080.0 pg/mL) than those without LNM (59.5 pg/mL, range: 12.5–1864.0 pg/mL); *p* < 0.05). Additionally, patients with LNM were more likely to present with multifocal lesions (24.1% vs. 2.3%; *p* < 0.01) and high-grade tumors (55.2% vs. 2.3%; *p* < 0.001). Preoperative ultrasonography detected suspicious lymph nodes in six patients with LNM (five in the lateral compartment and one in the central compartment), whereas none was detected in patients without LNM (*p* < 0.01). As presented in **Table 3**, parameters including age at diagnosis, sex, year of diagnosis, comorbidities, basal calcitonin level, basal CEA level, patients who underwent FNA-CT, FNA-CT value, tumor size, tumor extension, tumor multifocality, and initial surgical approach were comparable between patients with an excellent response and those with a biochemical incomplete response. In contrast, suspicious lymph nodes detected in preoperative ultrasonograms, LNM, AJCC clinical stage, and tumor grade were associated with the occurrence of biochemical incomplete response during postoperative follow-up (all *p* < 0.05). Given the strong correlations between suspicious lymph nodes detected in preoperative ultrasonograms and LNM, as well as the dependence of AJCC staging on lymph node status, only AJCC clinical stage and tumor grade were considered influencing factors for dynamic risk stratification during postoperative follow-up.

**Table 2:** Factors associated with lymph node metastasis in patients with micro-sMTC.

Variables	Without LNM (n = 44)	With LNM (n = 29)	*p*-value
Age at diagnosis (years)	47.3 ± 10.8	49.8 ± 12.8	0.3995
Sex			>0.999
Female	27 (61.4%)	17 (58.6%)	
Male	17 (38.6%)	12 (41.4%)	
Year of diagnosis			0.9307
2014–2019	6 (13.6%)	5 (17.2%)	
2020–2024	38 (86.4%)	24 (82.8%)	
Comorbidities			0.9307
With HT	6 (13.6%)	5 (17.2%)	
Without HT	38 (86.4%)	24 (82.8%)	
Basal calcitonin level (pg/mL)	59.5 (12.5–1864.0)	140.3 (13.1–7080.0)	<0.05
Basal CEA level (ng/mL)	3.3 (0.7–32.3)	4.0 (1.0–291.0)	0.3048
FNA-CT cases			0.1954
No	12 (27.3%)	13 (44.8%)	
Yes	32 (72.7%)	16 (55.2%)	
FNA-CT value ^a^ (pg/mL)	53426.0 (3654.0–200000.0) ^b^	28141.0 (3453.0–200000.0) ^c^	0.4180
Tumor size (mm)	7.0 (3.0–10.0)	7.0 (4.0–10.0)	0.5239
Tumor size category (mm)			0.0611
1.0–5.0	18 (40.9%)	5 (17.2%)	
6.0–10.0	26 (59.1%)	24 (82.8%)	
Suspicious lymph nodes identified using preoperative US			<0.01
No	44 (100.0%)	23 (79.3%)	
Yes	0 (0.0%)	6 (20.7%)	
Tumor extension			0.1442
Intrathyroid	39 (88.6%)	21 (72.4%)	
ETE	5 (11.4%)	8 (27.6%)	
Foci			<0.01
Solitary	43 (97.7%)	22 (75.9%)	
Multifocal	1 (2.3%)	7 (24.1%)	
Tumor grade			<0.001
Low	43 (97.7%)	13 (44.8%)	
High	1 (2.3%)	16 (55.2%)	

micro-sMTC, Sporadic medullary thyroid microcarcinoma; LNM, Lymph node metastasis; HT, Hashimoto thyroiditis; CEA, Carcinoembryonic antigen; FNA-CT, Calcitonin assays in fine-needle aspiration washout fluid; US, Ultrasonography; ETE, Extrathyroidal extension.

^a^Data from 53 micro-MTC nodules of 48 patients; ^b^Data from 33 micro-MTC nodules of 32 patients; ^c^Data from 20 micro-MTC nodules of 16 patients.

**Table 3:** Factors associated with dynamic risk stratification.

Variables	Excellent (n = 58)	Biochemical incomplete (n = 12)	*p*-value
Age at diagnosis (years)	49.2 ± 10.9	44.0 ± 13.5	0.230
Sex			>0.999
Female	35 (60.3%)	7 (58.3%)	
Male	23 (39.7%)	5 (41.7%)	
Year of diagnosis			0.386
2014–2019	8 (13.8%)	3 (25.0%)	
2020–2024	50 (86.2%)	9 (75.0%)	
Comorbidities			0.678
With HT	8 (13.8%)	2 (16.7%)	
Without HT	50 (86.2%)	10 (83.3%)	
Basal calcitonin level (pg/mL)	65.5 (12.5–7080.0)	68.8 (16.5–1410.0)	0.404
Basal CEA level (ng/mL)	3.3 (0.7–291.0)	3.0 (1.0–16.5)	0.932
FNA-CT cases			0.062
No	15 (25.9%)	7 (58.3%)	
Yes	43 (74.1%)	5 (41.7%)	
FNA-CT value ^a^ (pg/mL)	34222.0 (3453.0–200000.0) ^b^	30429.0 (9847.0–200000.0) ^c^	0.933
Tumor size (mm)	7.0 (3.0–10.0)	6.0 (3.0–10.0)	0.587
Tumor size category (mm)			>0.999
1.0–5.0	19 (32.8%)	4 (33.3%)	
6.0–10.0	39 (67.2%)	8 (66.7%)	
Suspicious lymph nodes detected using preoperative US			<0.05
No	56 (96.6%)	9 (75.0%)	
Yes	2 (3.4%)	3 (25.0%)	
Tumor extension			0.216
Intrathyroid	49 (84.5%)	8 (66.7%)	
ETE	9 (15.5%)	4 (33.3%)	
Foci			0.092
Solitary	54 (93.1%)	9 (75.0%)	
Multifocal	4 (6.9%)	3 (25.0%)	
Tumor grade			<0.001
Low	52 (89.7%)	4 (33.3%)	
High	6 (10.3%)	8 (66.7%)	
Initial surgical approach			0.442
Hemithyroidectomy	12 (20.7%)	1 (8.3%)	
Total thyroidectomy	46 (79.3%)	11 (91.7%)	
LND extent			0.136
Central LND	48 (82.8%)	7 (58.3%)	
Central and Lateral LND	10 (17.2%)	5 (41.7%)	
Lymph node status			<0.001
N0	42 (72.4%)	2 (16.7%)	
N1	16 (27.6%)	10 (83.3%)	
AJCC clinical stage			<0.001
I	42 (72.4%)	2 (16.7%)	
III	13 (22.4%)	7 (58.3%)	
IV	3 (5.2%)	3 (25.0%)	

HT, Hashimoto thyroiditis; CEA, carcinoembryonic antigen; FNA-CT, Calcitonin assays in fine-needle aspiration washout fluid; US, Ultrasonography; ETE, Extrathyroidal extension; LND, Lymph node dissection; AJCC, American Joint Committee on Cancer.

^a^Data from 53 micro-MTC nodules of 48 patients; ^b^Data from 47 micro-MTC nodules of 43 patients; ^c^Data from 6 micro-MTC nodules of 5 patients.

### Significance of FNA-CT in the management of sporadic micro-MTC

We explored the potential contribution of FNA-CT to the clinical management of micro-sMTC. As detailed in **Table 4**, 11 patients treated before 2020 did not undergo FNA-CT, whereas 48 out of 62 patients (77.4%) treated after 2020 underwent FNA-CT. The patients who underwent FNA-CT had significantly lower basal calcitonin levels (40.8 pg/mL [range: 12.5–7080.0 pg/mL] vs. 195.7 pg/mL [range: 27.7–1956.0 pg/mL]; *p* < 0.001) and basal CEA levels (2.9 ng/mL [range: 0.7–291.0 ng/mL] vs. 8.1 ng/mL [range: 1.0–30.5 ng/mL]; *p* < 0.01) than those who did not undergo FNA-CT. In addition, patients in the FNA-CT group had significantly smaller tumor size (6.0 mm [range: 3.0–10.0 mm] vs. 9.0 mm [range: 4.0–10.0 mm]; *p* < 0.001), a higher proportion of stage I disease (66.7% vs. 48.0%; *p* < 0.05), and a higher prevalence of low-grade MTC lesions (85.4% vs. 60.0%; *p* < 0.05) than those in the non-FNA-CT group. These findings suggest that FNA-CT has the potential to detect early micro-sMTC. Consequently, treatment strategies for micro-sMTC have been improved with the use of FNA-CT since 2020. Of the 48 patients who underwent FNA-CT after 2020, 13 patients (27.1%) underwent hemithyroidectomy. Preoperative evaluations of these 13 patients did not reveal any suspicious lymph nodes, each had a solitary malignant lesion, and no obvious capsule invasion was detected in preoperative ultrasonograms. The clinical pathological characteristics and follow-up information of these 13 patients are presented in **Supplementary Table 1** The median tumor size of these 13 patients was 4.0 mm (range: 3.0–5.0 mm). Four patients had a nodular goiter on the contralateral lobe, with FNA-CT values significantly lower than those of the MTC lesions. Final pathological results indicated that only one patient had one central LNM, and all MTC lesions were low-grade. At 6-months postoperatively, one patient exhibited biochemical incomplete response, whereas the remaining 12 had excellent responses until the last follow-up. Back to **Table 4**, until the latest follow-up, a higher proportion of patients who underwent FNA-CT showed an excellent response to initial surgery compared with those who did not (89.6% vs. 60.0%; *p* < 0.01), moreover, all three cases of structural incomplete response occurred in patients who did not undergo FNA-CT (12.0% vs. 0; *p* < 0.01).

**Table 4:** Comparison between patients who underwent FNA-CT and those who did not.

Variables	Without FNA-CT (n = 25)	With FNA-CT (n = 48)	*p*-value
Age at diagnosis (years)	48.6 ± 13.7	48.1 ± 10.7	0.878
Sex			>0.999
Male	15 (60.0%)	29 (60.4%)	
Female	10 (40.0%)	19 (39.6%)	
Year of diagnosis			<0.001
2014–2019	11 (44.0%)	0 (0.0%)	
2020–2024	14 (56.0%)	48 (100.0%)	
Comorbidities			>0.999
Without HT	21 (84.0%)	41 (85.4%)	
With HT	4 (16.0%)	7 (14.6%)	
Initial presentation			0.067
Asymptomatic suspicious thyroid nodule detected using US	21 (84.0%)	46 (95.8%)	
Enlarged cervical lymph nodes	4 (16.0%)	1 (2.1%)	
Hyperthyroidism	0 (0.0%)	1 (2.1%)	
Basal calcitonin level (pg/mL)	195.7 (27.7–1956.0)	40.8 (12.5–7080.0)	<0.001
Basal CEA level (ng/mL)	8.1 (1.0–30.5)	2.9 (0.7-291.0)	<0.01
FNA-CT value (pg/mL)	/	342222.0 (3453.0– 200000.0) ^a^	/
Suspicious lymph nodes identified using preoperative US			0.172
No	21 (84.0%)	46 (95.8%)	
Yes	4 (16.0%)	2 (4.2%)	
FNAC ^b^			0.502
I	2 (8.0%)	8 (14.0%)	
II	0	4 (7.0%)	
III	13 (52.0%)	30 (52.6%)	
IV	2 (8.0%)	3 (5.3%)	
V-VI	8 (32.0%)	12 (21.1%)	
Tumor size (mm)	9.0 (4.0–10.0)	6.0 (3.0–10.0)	<0.001
Tumor size category (mm)			<0.001
1.0–5.0	1 (4.0%)	22 (45.8%)	
6.0–10.0	24 (96.0%)	26 (54.2%)	
Tumor extension			0.347
Intrathyroid	19 (76.0%)	41 (85.4%)	
ETE	6 (24.0%)	7 (14.6%)	
Foci			>0.999
Solitary	22 (88.0%)	43 (89.6%)	
Multifocal	3 (12.0%)	5 (10.4%)	
Lymph node status			0.195
N0	12 (48.0%)	32 (66.7%)	
N1	13 (52.0%)	16 (33.3%)	
Distant metastasis			/
M0	25 (100.0%)	49 (100.0%)	
M1	0	0	
AJCC clinical stage			<0.05
I	12 (48.0%)	32 (66.7%)	
III	7 (28.0%)	14 (29.2%)	
IV	6 (24.0%)	2 (4.2%)	
Tumor grade			<0.05
Low	15 (60.0%)	41 (85.4%)	
High	10 (40.0%)	7 (14.6%)	
Initial surgical approach			<0.05
Hemithyroidectomy	0 (0.0%)	13 (27.1%)	
Total thyroidectomy	25 (100.0%)	35 (72.9%)	
LND extent			<0.001
Central LND	11 (44.0%)	45 (93.8%)	
Central and Lateral LND	14 (56.0%)	3 (6.2%)	
Postoperative complications			/
Transient hypoparathyroidism	14 (56.0%)	16 (33.3%)	
Transient RLN injury	0	2 (4.2%)	
Permanent hypoparathyroidism	0	0	
Permanent RLN injury	0	0	
Chylous leakage	1 (4.0%)	0	
Horner syndrome	1 (4.0%)	0	
Follow-up duration (months)	51.0 (7.0–124.0)	27.5 (6.0–58.0)	<0.001
Response to initial surgery			<0.01
Excellent	15 (60.0%)	43 (89.6%)	
Biochemical incomplete	7 (28.0%)	5 (10.4%)	
Structural incomplete	3 (12.0%)	0	

FNA-CT, Calcitonin assays in fine-needle aspiration washout fluid; HT, Hashimoto thyroiditis; CEA, Carcinoembryonic antigen; US, Ultrasonography; FNAC, Fine-needle aspiration cytology; ETE, Extrathyroidal extension; AJCC, American Joint Committee on Cancer; LND, Lymph node dissection; RLN, Recurrent laryngeal nerve.

^a^Data from 53 micro-MTC nodules of 48 patients; ^b^Data from 82 nodules of 73 patients.

### Progression-free survival analysis

**Figure 2:**
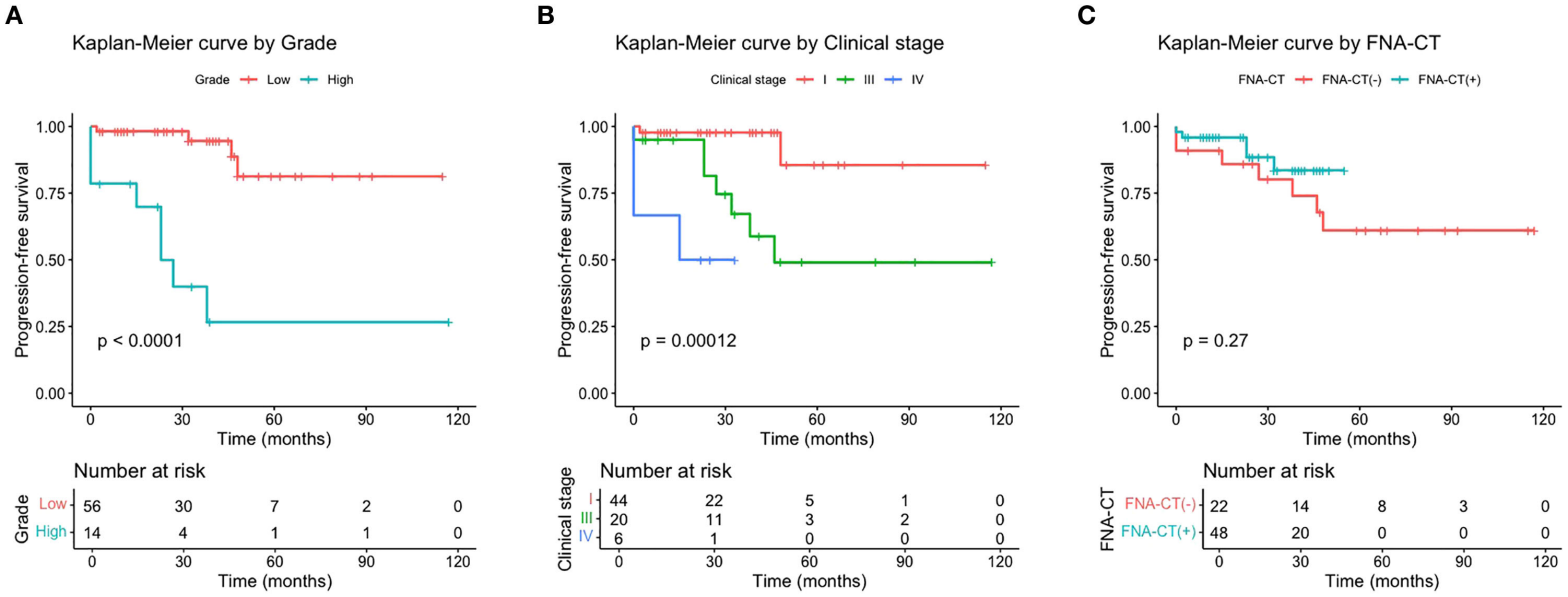
Progression-free survival curves by tumor grade **(A)**, AJCC clinical stage **(B)**, and FNA-CT **(C)**.

Starting from 3 months postoperatively, the median time to the occurrence of a biochemical incomplete response was 23.0 months (range: 0.0–48.0 months) in the 12 patients. Until the last follow-up, no structural incomplete responses were observed for them. Univariate and multivariate Cox proportional hazards regression analyses indicated that high-grade tumors (HR = 7.111, 95% CI: 1.745–28.980; *p* < 0.01) and advanced clinical stages (HR = 11.665, 95% CI: 1.455–93.900; *p* < 0.05) were independent risk factors for poor PFS (**Supplementary Table 2**). Kaplan–Meier survival curves were generated to visualize these results (**Figures 2A–C**). Unexpectedly, the Kaplan–Meier survival curve analysis showed that the use of FNA-CT did not significantly improve patients’ PFS (**Figure 2C**). The details of the three patients with structural incomplete responses are listed in **Supplementary Table 3**. All three patients experienced local recurrence at 39, 32, and 14 months after initial treatment and further neck exploration with LND was performed to achieve local disease control. At the last follow-up, all three patients achieved excellent responses to the second surgery.

## Discussion

The detection rate of micro-sMTC is expected to increase because of the growing use of FNA-CT in recent years (22, 29). However, the clinicopathological characteristics, treatment strategies, and long-term prognosis remain ambiguous owing to limited and inconsistent evidence. We conducted this 10-year retrospective study to show that although the prognosis of micro-sMTC was generally favorable, aggressive features such as LNM, high grade, multifocality, ETE, and advanced clinical stage should not be overlooked. These factors were associated with LNM, postoperative dynamic risk stratification, and PFS. FNA-CT facilitated earlier detection of micro-sMTC and may have the potential to locate MTC lesions within multinodular lesions when hemithyroidectomy is considered.

Regional LNM is not uncommon and is associated with poor prognosis for MTC, with a reported prevalence ranging from 12.0% to 44.8% (5, 8, 30, 31). However, these findings may be influenced by biases inherent in population-based studies that did not differentiate between hereditary and sporadic micro-MTC cases (8, 31). Our study exclusively included micro-sMTC, showing that 29 patients (39.7%) had regional LNM, including 8 patients (11.0%) with lateral neck lymph node metastasis (LLNM). Of the 8 patients with LLNM, 5 patients had suspicious lymph node lesions detected using ultrasonography preoperatively, and 6 patients had basal calcitonin levels over 200 pg/mL. Therefore, the decision to perform lateral LND was largely based on the clinical and/or ultrasonographic evidence of LNM and markedly increased basal calcitonin levels in this study. In other words, prophylactic LLND should be performed with caution, as there is no consensus on its association with improved long-term prognosis (32). Basal calcitonin level, suspicious lymph nodes detected in preoperative ultrasonograms, and multifocal and high-grade lesions were identified as influencing factors of LNM in this study, which is consistent with previous studies (5, 8, 30). Basal calcitonin level is associated with tumor burden and is recommended by European Society for Medical Oncology guidelines as a reference for determining the extent of LND (19). Nevertheless, preoperative neck ultrasonography has a limited role in the evaluation of central LNM, and multifocality and tumor grade can only be determined postoperatively, limiting their utility as predictive markers for LNM before surgery. However, these two postoperative pathological features can be used as vital references for determining the intensity and frequency of follow-up postoperatively.

Although the outcomes for patients with micro-sMTC are typically good, postoperative dynamic risk stratification assessment, which is strongly recommended for differentiated thyroid carcinoma, should also be applied to each patient with micro-sMTC. Some studies have confirmed that dynamic risk stratification assessment can provide useful prognostic information in patients with MTC (33–35). In our study, after a median follow-up of 34.0 months, 79.5% of patients achieved an excellent response, 16.4% of patients had a biochemical incomplete response, and 4.1% of patients exhibited a structural incomplete response. These outcomes are marginally better than those reported in a recent study, and we speculate that the differences may be attributed to the lower incidence of LNM, lower basal calcitonin levels, and earlier clinical stages observed in our cohort (30). High-grade micro-MTC was associated with a structural incomplete response in a study by Kesby (5). In line with this finding, all three patients with structural incomplete responses in our cohort had high-grade tumors, underscoring the importance of routinely reporting tumor grade in pathological reports of micro-MTC to guide appropriate follow-up and subsequent treatment strategies. Admittedly, the favorable prognosis and small number of recurrence events in this study prevented a comprehensive evaluation of the role of tumor grade in disease-free survival, highlighting the need for future studies with larger sample sizes to validate these findings.

Surgery is currently regarded as the only curative option for patients with MTC, whereas targeted therapies, such as vandetanib, cabozantinib, selpercatinib, and pralsetinib, are indicated for patients with metastatic or advanced disease. According to established guidelines, TT with central LND has been a widely accepted surgical approach for patients with micro-MTC. Advances in diagnostic techniques, such as the increasing clinical application of FNA-CT, have led to an increasing detection rate of micro-MTC. Concurrently, increasing awareness of thyroid function preservation and concerns regarding postoperative complications have led to an increasing demand for less extensive surgical approaches, such as hemithyroidectomy. Importantly, appropriate selection criteria should be established for patients undergoing hemithyroidectomy. The JAES guideline recommends hemithyroidectomy for sporadic MTC cases where the lesion is confined to a single thyroid lobe (16). A recent European study used ex-post criteria for the presurgical localization of MTC nodules to demonstrate the feasibility of hemithyroidectomy for treating patients who were RET-negative with a suspicion of MTC (36). Similarly, a multi-institutional study by Mao YV indicated that hemithyroidectomy could be considered in patients with sporadic MTC without contralateral ultrasonography findings, with no need for further surgery if calcitonin levels were undetectable (37). Another nationwide large-scale cohort study from China also demonstrated that hemithyroidectomy could be an alternative option that did not compromise prognosis while avoiding additional complications associated with TT in patients with small unilateral sMTC (≤ 2 cm) (38, 39). Park H suggested that hemithyroidectomy could be considered in patients with an MTC diameter of ≤ 2.5 cm, clinically negative lymph nodes, and a basal calcitonin level of ≤ 250 pg/mL (40). In the present study, all 13 patients who underwent hemithyroidectomy had MTC lesions with a maximum diameter of ≤ 5.0 mm, clinically negative lymph nodes, and a median basal calcitonin level of 40.3 pg/mL (range: 12.5–77.8 pg/mL). Final pathological examination revealed that only one patient had a single central LNM, one patient had minimal ETE, and all 13 patients had low-grade tumors. Follow-up data revealed that 12 patients (92.3%) had an excellent response to the initial surgery, whereas only one patient (7.7%) exhibited a biochemical incomplete response 6 months postoperatively. Particularly, 4 out of the 13 patients had bilateral thyroid nodules, with a suspicious malignant nodule on one side and a nodular goiter on the contralateral side on ultrasonograms. In these four cases, FNAC and FNA-CT were performed on both nodules to exclude the presence of occult MTC lesions in the contralateral lobe, thereby supporting the decision to proceed with hemithyroidectomy. These findings suggest that FNA-CT holds promise for localizing MTC lesions and may serve as a valuable tool for selecting appropriate candidates for hemithyroidectomy. However, large-scale prospective studies are warranted to validate these preliminary results.

Previous studies, including our recent study, have demonstrated that FNA-CT is an auxiliary and cost-effective method for diagnosing MTC, exhibiting high sensitivity and specificity (9, 22, 23). However, the benefit of FNA-CT use on the clinical management of micro-MTC remains unclear. Although comparative analysis showed that patients who underwent FNA-CT were diagnosed with micro-sMTC at a relatively earlier stage than those who did not, this earlier detection did not translate into a significant improvement in PFS in the present study. This finding may be attributed to the typically favorable prognosis of micro-sMTC, limited sample size, and relatively short follow-up duration in our cohort. Furthermore, although all three patients with structural incomplete responses did not undergo FNA-CT, this finding did not necessarily indicate that the use of FNA-CT would have improved their prognosis. In these three patients, the preoperative basal serum calcitonin levels were already markedly elevated, making additional confirmation via FNA-CT unnecessary. The prognoses of these three patients were largely based on the inherent tumor burden rather than the use of FNA-CT at that stage. However, if FNA-CT had been applied when the tumor burden was low, earlier detection may have contributed to a better prognosis. Nevertheless, concerns regarding the use of FNA-CT should be acknowledged, including variable methods for measuring calcitonin and different cutoff values for MTC diagnosis across studies, as well as potential false positives from C-cell hyperplasia and peripheral blood contamination. Despite these concerns, the ATA guidelines have recommended the use of FNA-CT for the preoperative evaluation of patients with modestly elevated basal calcitonin levels, with a Grade B recommendation (1). We believe that the accumulation of stronger evidence-based data will lead to increased clinical adoption of FNA-CT, not only as a diagnostic tool but also as a reference for guiding surgical decision-making.

Several limitations of this study should be declared, including its retrospective design, small sample size, relatively short follow-up duration, and a potentially overestimated PFS resulting from some patients independently extending their follow-up intervals. Some patients were followed up in external hospitals, and this may introduce potential biases in calcitonin assay methodology and ultrasound examination. Moreover, the degree of connective tissue proliferation and thyroid capsule infiltration, two important indicators for determining the extent of surgery, were not routinely evaluated using intraoperative frozen section examination in our center, which may have resulted in insufficient resection in some patients who underwent hemithyroidectomy. In addition, grouping by “2014–2019 vs. 2020–2024” coincides with several contemporaneous changes after 2020, such as the introduction of FNA-CT, greater ultrasound screening intensity, evolving surgical strategies, and standardized pathology grading. These era effects could increase detection yield, advance the timing of diagnosis (lead-time), shorten follow-up, and induce stage migration, thereby potentially inflating PFS and undermining the interpretation that “FNA-CT provides no PFS benefit.” Therefore, well-designed prospective studies with larger sample sizes, extended follow-up durations, and stronger control for calendar time and practice changes are warranted to comprehensively investigate the pathophysiology, natural history, treatment strategies, follow-up protocols, and prognosis of micro-sMTC, as well as to further clarify the prognostic value of FNA-CT in these patients.

In conclusion, this study suggests that FNA-CT enhances the early detection of micro-sMTC, a disease that typically has a favorable prognosis. However, aggressive features such as LNM, multifocality, ETE, and high tumor grade may still be present. Prognostic factors, including high tumor grade and advanced clinical stage, are associated with shorter PFS and may help guide postoperative surveillance strategies. Additionally, FNA-CT has potential value in selecting appropriate candidates for hemithyroidectomy.

## Data Availability

The raw data supporting the conclusions of this article will be made available by the authors, without undue reservation.
