# Supplementary material for: Clinicopathological characteristics, prognosis, and the significance of FNA-CT in sporadic medullary thyroid microcarcinoma: a 10-year retrospective study
Source: Front Endocrinol (Lausanne). 2025 Oct 15;16:1676241. doi: 10.3389/fendo.2025.1676241 (PMC12568346; doi:10.3389/fendo.2025.1676241)
Supplement: Supplementary file 1 [file Table1.docx]

Supplementary Table 1 Information on the 13 patients who underwent hemithyroidectomy.

| Case | Age at diagnosis (years) | Sex | Basal calcitonin level (pg/mL) | Suspicious lymph node identified using preoperative US | Tumor size (mm) | FNA-CT for MTC (pg/mL) | Goiter on the contralateral lobe | FNA-CT for goiter (pg/mL) | LNM | ETE | Tumor grade | AJCC clinical stage | Follow-up time (months) | Response |
| --- | --- | --- | --- | --- | --- | --- | --- | --- | --- | --- | --- | --- | --- | --- |
| 1 | 43 | F | 37.0 | No | 3.0 | 9847.0 | No | / | No | No | Low | I | 27 | Biochemical incomplete |
| 2 | 45 | F | 14.7 | No | 5.0 | 9858.0 | No | / | No | No | Low | I | 27 | Excellent |
| 3 | 58 | F | 40.3 | No | 5.0 | 200000.0 | No | / | Yes | Yes | Low | III | 11 | Excellent |
| 4 | 43 | M | 12.5 | No | 3.0 | 8949.0 | Yes | 0.5 | No | No | Low | I | 11 | Excellent |
| 5 | 58 | M | 33.6 | No | 4.0 | 17890.0 | No | / | No | No | Low | I | 48 | Excellent |
| 6 | 51 | F | 54.0 | No | 5.0 | 145456.0 | No | / | No | No | Low | I | 35 | Excellent |
| 7 | 38 | F | 32.0 | No | 4.0 | 200000.0 | No | / | No | No | Low | I | 35 | Excellent |
| 8 | 53 | F | 13.4 | No | 4.0 | 34567.0 | Yes | 245.0 | No | No | Low | I | 45 | Excellent |
| 9 | 54 | F | 76.6 | No | 4.0 | 57443.0 | No | / | No | No | Low | I | 28 | Excellent |
| 10 | 33 | F | 54.5 | No | 4.0 | 200000.0 | Yes | 43.0 | No | No | Low | I | 12 | Excellent |
| 11 | 57 | M | 65.3 | No | 5.0 | 11029.0 | No | / | No | Yes | Low | I | 25 | Excellent |
| 12 | 47 | F | 68.2 | No | 5.0 | 43011.0 | No | / | No | No | Low | I | 14 | Excellent |
| 13 | 49 | M | 77.8 | No | 4.0 | 55585.0 | Yes | 0.5 | No | No | Low | I | 11 | Excellent |

US: Ultrasonography; FNA-CT: Calcitonin assays in fine-needle aspiration washout fluid; MTC: Medullary thyroid carcinoma; LNM: Lymph node metastasis; ETE: Extrathyroidal extension; AJCC: American Joint Committee on Cancer; F: Female; M: Male.

Supplementary Table 2 Identification of predictors associated with PFS.

| Variables | Univariate Cox regression analyses | |  | Multivariate Cox regression analyses | |
| --- | --- | --- | --- | --- | --- |
|  | HR (95% CI) | *p*-value |  | HR (95% CI) | *p*-value |
| Age at diagnosis | 0.958 (0.909–1.009) | 0.102 |  |  |  |
| Sex |  |  |  |  |  |
| Female | Reference |  |  |  |  |
| Male | 1.274 (0.402–4.039) | 0.680 |  |  |  |
| Year of diagnosis |  |  |  |  |  |
| 2014–2019 | Reference |  |  |  |  |
| 2020–2024 | 1.440 (0.356–5.821) | 0.609 |  |  |  |
| Comorbidities |  |  |  |  |  |
| Without HT | Reference |  |  |  |  |
| With HT | 1.009 (0.219–4.652) | 0.991 |  |  |  |
| Basal calcitonin level (pg/mL) | 1.000 (0.999–1.001) | 0.946 |  |  |  |
| Basal CEA level (ng/mL) | 0.987 (0.920–1.059) | 0.715 |  |  |  |
| FNA-CT cases |  |  |  |  |  |
| No | Reference |  |  |  |  |
| Yes | 0.516 (0.157–1.698) | 0.276 |  |  |  |
| FNA-CT ^a^ value (pg/mL) | 1.000 (1.000–1.000) | 0.841 |  |  |  |
| Tumor size (mm) | 0.841 (0.633–1.118) | 0.232 |  |  |  |
| Tumor size category (mm) |  |  |  |  |  |
| 1.0–5.0 | Reference |  |  |  |  |
| 6.0–10.0 | 0.608 (0.172–2.143) | 0.438 |  |  |  |
| Tumor extension |  |  |  |  |  |
| Intrathyroid | Reference |  |  |  |  |
| ETE | 2.551 (0.760–8.557) | 0.129 |  |  |  |
| Foci |  |  |  |  |  |
| Solitary | Reference |  |  |  |  |
| Multifocal | 3.646 (0.985–13.504) | 0.053 |  |  |  |
| Tumor grade |  |  |  |  |  |
| Low | Reference |  |  | Reference |  |
| High | 12.647 (3.715–43.057) | <0.001 |  | 7.111 (1.745–28.980) | <0.01 |
| AJCC clinical stage |  |  |  |  |  |
| I | Reference |  |  | Reference |  |
| III | 7.050 (1.462–33.987) | <0.05 |  | 2.811 (0.477–16.570) | 0.25343 |
| IV | 24.350 (3.792–156.361) | <0.001 |  | 11.665 (1.455–93.900) | <0.05 |
| Initial surgical approach |  |  |  |  |  |
| Hemithyroidectomy | Reference |  |  |  |  |
| Total thyroidectomy | 1.613 (0.203–12.831) | 0.651 |  |  |  |
| LND extent |  |  |  |  |  |
| Central LND | Reference |  |  |  |  |
| Central and Lateral LND | 2.784 (0.880–8.802) | 0.081 |  |  |  |

PFS: Progression-free survival; HT: Hashimoto thyroiditis; CEA: Carcinoembryonic antigen; FNA-CT: Calcitonin assays in fine-needle aspiration washout fluid; ETE: Extrathyroidal extension; LND: Lymph node dissection; AJCC: American Joint Committee on Cancer

^a^Data from 53 micro-MTC nodules of 48 patients.

Supplementary Table 3 Information on the three patients with structural incomplete response.

| Variables | Case 1 | Case 2 | Case 3 |
| --- | --- | --- | --- |
| Age at diagnosis (years) | 67 | 51 | 26 |
| Sex | Female | Male | Female |
| Year of diagnosis | 2021 | 2022 | 2023 |
| Comorbidities | CVD | None | None |
| Basal calcitonin level (pg/mL) | 249.7 | 1956.0 | 272.3 |
| Basal CEA level (ng/mL) | 8.48 | 21.2 | 6.24 |
| FNA-CT | No | No | No |
| Tumor size (mm) | 7.0 | 7.0 | 9.0 |
| Suspicious lymph nodes detected using preoperative US | No | Yes | No |
| Initial surgical approach | TT + central and lateral LND | TT + central and lateral LND | TT + central and lateral LND |
| Tumor extension | No | No | No |
| Foci | Multifocal | Solitary | Solitary |
| Tumor grade | High | High | High |
| LNM | Yes | Yes | Yes |
| AJCC clinical stage | III | IV | IV |
| Postoperative complications | Transient hypoparathyroidism | Transient hypoparathyroidism | Transient hypoparathyroidism |
| Follow-up duration (months) | 50 | 38 | 26 |
| Time to structural incomplete response (months) | 39 | 32 | 14 |
| Site of recurrence | Local | Local | Local |
| Further surgery | Yes | Yes | Yes |
| Response at last follow-up | Excellent | Excellent | Excellent |

CVD: Cardiovascular diseases; CEA: Carcinoembryonic antigen; FNA-CT: Calcitonin assays in fine-needle aspiration washout fluid; US: Ultrasonography; TT: Total thyroidectomy; LND: Lymph node dissection; LNM: Lymph node metastasis; AJCC: American Joint Committee on Cancer.
